# Exploring women’s experiences of care during hospital childbirth in rural Tanzania: a qualitative study

**DOI:** 10.1186/s12884-024-06396-0

**Published:** 2024-04-19

**Authors:** Emmy Metta, Regine Unkels, Lilian Teddy Mselle, Claudia Hanson, Helle Molsted Alvesson, Fadhlun M. Alwy Al-beity

**Affiliations:** 1 Department of Behavioural Sciences, School of Public Health and Social Sciences, Muhimbili University of Health and Allied Sciences, Dar es Salaam, Tanzania; 2 Department of Global Public Health, Karolinska Institutet, Stockholm, Sweden; 3 Department of Clinical Nursing, School of Nursing, Muhimbili University of Health and Allied Sciences, Dar es Salaam, Tanzania; 4 Department of Obstetrics/Gynaecology, School of Clinical Medicine, Muhimbili University of Health and Allied Sciences, Dar es Salaam, Tanzania

**Keywords:** Women’s experiences, Childbirth, Quality improvement, Rural health services, Respectful care, Birth companionship, Co-design, Tanzania

## Abstract

**Background:**

Women’s childbirth experiences provide a unique understanding of care received in health facilities from their voices as they describe their needs, what they consider good and what should be changed. Quality Improvement interventions in healthcare are often designed without inputs from women as end-users, leading to a lack of consideration for their needs and expectations. Recently, quality improvement interventions that incorporate women’s childbirth experiences are thought to result in healthcare services that are more responsive and grounded in the end-user’s needs.

**Aim:**

This study aimed to explore women’s childbirth experiences to inform a co-designed quality improvement intervention in Southern Tanzania.

**Methods:**

This exploratory qualitative study used semi-structured interviews with women after childbirth (*n* = 25) in two hospitals in Southern Tanzania. Reflexive thematic analysis was applied using the World Health Organization’s Quality of Care framework on experiences of care domains.

**Results:**

Three themes emerged from the data: (1) Women’s experiences of communication with providers varied (2) Respect and dignity during intrapartum care is not guaranteed; (3) Women had varying experience of support during labour. Verbal mistreatment and threatening language for adverse birthing outcomes were common. Women appreciated physical or emotional support through human interaction. Some women would have wished for more support, but most accepted the current practices as they were.

**Conclusion:**

The experiences of care described by women during childbirth varied from one woman to the other. Expectations towards empathic care seemed low, and the little interaction women had during labour and birth was therefore often appreciated and mistreatment normalized. Potential co-designed interventions should include strategies to (i) empower women to voice their needs during childbirth and (ii) support healthcare providers to have competencies to be more responsive to women’s needs.

**Supplementary Information:**

The online version contains supplementary material available at 10.1186/s12884-024-06396-0.

## Background

Childbirth represents a vulnerable time in a woman’s life, with unique physical, psychological and emotional needs [1, 2]. Positive childbirth experiences enhance the feeling of autonomy, boost mothers’ confidence, and enhance maternal-newborn bonding, leading to maternal well-being [3–5]. Negative childbirth experiences undermine a woman’s confidence and predispose women to postpartum mental health disorders, including anxiety and depression. Such adverse experiences reduce women’s satisfaction with the healthcare system and may deter women from seeking care for future pregnancies [6].

A recent systematic review of childbirth experiences in sub-Saharan Africa reported sub-optimal communication and emotional support as standard practice in public health facilities [3]. Poor childbirth experiences, including mistreatment, neglect and abandonment, and use of physical, verbal, and emotional violence at facility levels, are widely reported in low and middle-income settings, including Tanzania [7–12]

Quality improvement interventions in maternity care are often designed along a structured framework historically inspired by Donabedian’s approach [13] by professionals for end-users. Few quality interventions have been tailored to improve specific areas of quality care, such as respectful maternity care, patient-centered care, and birth companionship [14–17]. These interventions, however, were primarily conducted in research settings and were not always informed by women’s experiences. There is building evidence that healthcare interventions grounded in the end-users need increase the potential for meaningful implementation adoption, sustainability, and scale-up [18, 19].

While there is a large body of evidence documenting poor childbirth experiences in different sub-Saharan countries, few of these are taken further during the design of interventions to improve peripartum care in line with end-user needs [19, 20]. This study aims to explore childbirth experiences in Tanzania’s rural settings with the potential to integrate these experiences to co-design an intrapartum quality improvement intervention.

## Methods

The research presented formed the initial phase of the co-design process for an intervention to intrapartum quality improvement known as “Action Leveraging Evidence to Reduce perinatal Mortality and Morbidity (ALERT) in four sub-Saharan African countries: Benin, Malawi, Tanzania and Uganda” [21].

### Study design

An exploratory qualitative study used in-depth interviews with women who gave birth in selected hospitals in rural Tanzania.

### Study setting

The study was conducted in the Southern regions of Tanzania. In the past, this region was considered to have higher maternal mortality than the rest of the country; however, more recent information is not available [22, 23]. The country’s maternal mortality ratio is 238 deaths per 100,000 live births and 238 deaths per 100,000 live births [uncertainty intervals 174–381] [24]. The area has a low number of skilled providers, 6.4 and 4.4 per 10,000 population for Lindi and Mtwara, respectively, lower than 23 providers per 10,000 population as recommended by WHO. Approximately 96% of women in the region deliver in health facilities [23].

The Tanzania health system has a district hospital as a first referral level, most owned by the government. Private hospitals are designated referral hospitals in a few areas with no government-owned hospitals and receive a government subsidy for operational costs [25]. By policy, healthcare services for pregnant women and newborns are free of cost in the country; however, women and their families usually incur out-of-pocket expenditures to cover medicines and consumables [26–28].

The Tanzania ALERT study is conducted in four hospitals purposefully selected to represent the typical rural Tanzania healthcare setting, including government and private-not-for-profit hospitals [21]. Additional hospital selection criteria were availability of obstetric services including operative deliveries, blood transfusion and neonatal units and annual number of births to be more than 2500 [21]. Hospital names are not included to protect confidentiality of participants. One of the four hospitals was owned by a faith-based organization where women and their families pay a modest fee for the services.

All hospitals received women from nearby communities and referrals from lower-level health facilities. Deliveries in the two hospitals, representative of typical district and referral hospitals in rural Tanzania, were conducted by nurse-midwives, most of them trained in local nursing schools. In the study hospitals maternity care was provided by nurse-midwives of certificate and diploma-level only.

Once pregnant women start labour, they are escorted by family members to the maternity unit. Women stay in the facility for 24–72 h after childbirth. As a standard practice in many facilities in the country, family members were not allowed to be in the labour ward, they stayed outside and waited for information. The exception was in the western part of the country, where implementation research was conducted to incorporate birth companionship in public health facilities [17]. The reported positive findings led to the Ministry of Health advocating for birth companionship while acknowledging infrastructural challenges that may hinder the practice at facility levels. Almost all regions including the southern region have not yet implemented birth companionship.

### Sampling and participant recruitment

The sample size decision was reached following Malterud K et al. (2016) concept of information power where researchers need to consider the breadth of study aim, sample specificity, quality of dialogue during data collection, use of theory and analysis strategy [29]. To gain a breath of information on childbirth experiences the study purposely recruited women with varying characteristics such as different modes of delivery, parity, referrals and birth outcomes. Women who were admitted after childbirth were excluded.

The postnatal ward midwife in-charge in consultation with the research team identified potential participants and introduced them to the researchers. The recruitment, consenting, and interviews were done during the discharge process of the woman from the hospital. The discharge process took a few hours, thus accommodating the interviews.

### Data collection

The interview guide (Additional file 1) was informed by the literature and was structured around communication, perceptions of respect and support given during childbirth. Pilot interviews outside the study region secured guide’s comprehension and length. Feedback from the pilot was used for wording and restructuring some of the questions for clarity and understanding.

A team of eight researchers with backgrounds in anthropology, obstetrics/gynaecology, midwifery and sociology conducted the interviews in Kiswahili, the National language well-spoken by almost all Tanzanians. All members of the team had contextual and clinical expertise. During data collection, the team met daily virtually or in-person to debrief. The interviews were face to face on one-to-one basis and were conducted in quiet, vacant rooms within the hospital to ensure privacy and confidentiality. Interviews lasted 35 – 50 min and were audio recorded after consent. Each team member conducted 1–2 interviews a day depending on availability of participants.

### Analysis

Interviews were transcribed verbatim and later translated into English. Transcripts were quality-checked by EM and FAA before being imported to MAXQDA version 2020 software to facilitate management and the analysis process.

Analysis was informed by the WHO Quality of Care framework, where the care process incorporates both the health outcomes and the individual experiences of receiving care. For quality childbirth care, there are three domains: effective communication, respectful and dignified care and availability of support including emotional well-being [30]. The domains and their indicators are presented in the Supplementary Figure.

All authors were familiar with the data during the debriefing meetings. Reflexive thematic analysis was used for analysis [31]. EM and FAA were immersed in the data, searching for clusters of meanings and patterns. This was followed by the generation of data-driven codes. Different codes were sorted into potential themes, and collated the relevant coded text into the identified themes. Themes were further refined for clarity and were linked with the WHO domains on experiences of quality of care to aid their presentation and interpretation [30, 32]. The last step was the writing, where we described the findings based on the WHO experiences of care concepts and included women’s accounts within and across the presented themes.

### Ethical issues

The study obtained ethical clearance from the *Muhimbili University of Health and Allied Sciences* institutional review board (MUHAS-REC-04-2020-118), and the *National Institute for Medical Research* Ethics Committee (NIMR/HQ/R.8a/Vol IX/3493). Each study participant provided written informed consent after receiving detailed information about the study in Kiswahili. There were no personal identifiers collected in the study. Women who had adverse outcomes were linked to counselling services through the existing hospital system.

## Results

Of the twenty-five women interviewed, most were between 18–34 years of age, completed primary school and were farmers. Participants mode of delivery and birth outcomes are shown in Table 1.

**Table 1:** Demographic characteristics of women interviewed (*N* = 25)

Participant characteristics	Number of respective participants
**Age group**	
< *18 years*	1
*18–34 years*	20
> *35 years*	4
**Median Age**	26 years
**Education level attained**	
*Did not complete primary*	2
*Completed primary*	16
*Secondary and above*	7
**Occupation**	
*Farmer*	13
*Petty trader*	8
*Manual skilled*	2
*Profession trained and formal employment*	2
**Mode of delivery**	
*Spontaneous vaginal delivery*	15
*Caesarian section*	9
*Assisted vaginal delivery-Lower vacuum extraction*	1
**Birth outcomes**	
*Alive and well*	1
*Admitted*	6
*Stillbirth*	1

The women’s experiences of childbirth are described based on the domains of the WHO framework on quality of care for maternal and new-born health: 1) Women’s experiences of communication with providers varied 2) Respect and dignity during childbirth is not guaranteed 3) Women had varying experience of support during labour. These larger themes included two to four subthemes as indicated on Table 2.

**Table 2:** Themes, sub-themes and selected quotes

Theme	Sub-theme	Some selected quotes
1. Women’s experiences of communication with providers varied	a. Some women experienced good communication	“As I entered here, the nurse said she has communicated with the facility where I come from…that she knows about me. I was relieved that they were expecting me” (Facility2_ IDIM12)
	b. Women received fragmented, unclear and one-way information	*“When they told me that the placenta is coming before the baby ….I did not understand in deep as what may be the result of the situation…..” (Facility1_IDI_M11)*
2. Respect and dignity during intrapartum care is not guaranteed	a. Some women were handled with respect	“*They liked me…I truly felt good because every time I had a problem, they attended to me.” (Facility2_IDI_M6)*
	b. Experiences of mistreatment were common	*“A*s she examined me I felt pain. *I started to complain. When I complained that nurse said, “The more you complain, the more you delay yourself, do you get me?” she was speaking with disrespect somehow.” (Facility2_IDI_M_9)*
	c. Women justified and normalized acts of mistreatment	*“Providers work in a difficult environment with many deliveries, and we women behave differently; sometimes, they (providers) react and need to be to that. Also, everyone has their own nature; some people are polite, and others are not. You take them as they are.”. [Facility2_IDI_M3]*
3. Women had varying experience of support during labour	a. Women appreciated being supported during labour	*“So, they (providers) keep encouraging you to push; they tell you that if you delay, the baby will fail to breathe; in that way, a mother becomes motivated, fearing to lose a baby. [a little laugh]. So she will be trying her best.” (Facility1_ IDI_M1)*
	b. Providers’ responsiveness and empathy differed from one woman to another	*“The male providers are always humble … If you complain, they say ‘pole’, they tell you to breathe, and you get relief a bit. It felt good to have such a person” (Facility1_IDI_M11)*
	c. Women’s perceptions and experiences with birth companionship varied	*“I would feel happy and grateful if my mother-in-law was allowed to be with me during labour.”(Facility_2 IDI_M10)* *“(laughter)… for labour process; it does not matter who is there with you… it is only you. Even if your father, mother, or brother came, they could not help; he looks at you….it is your struggle, eh!…” (Facility_2 IDI_M3)*

### Women’s experiences of communication with providers varied

Women encountered positive and negative experiences when communicating with health providers, as shown in the following.

#### Some women experienced good communication

Several women reported having good interactions with healthcare providers throughout the labour process. Women spontaneously reported feeling engaged as healthcare providers allowed them to ask questions and listen to their concerns.

*“Before he ruptured the membrane, he told me what he was going to do, and I said OKAY. Afterwards, he told me to continue resting, and that I will deliver within a short time.” (Facility 1 IDI_M11)*

#### Women received fragmented unclear and one-way information

Other women reported bad experiences as they interacted with healthcare providers. Some reported not being asked for consent during physical assessments and not being informed of the findings from the assessment. As one woman narrates her experience:

*“She (a provider) came and assessed me. She wore gloves, then she was doing this and that (gesturing with her hand) while I was in pain… She did not tell me anything, though… I wished to know, but it was just like that…” (Facility 2_IDI_M10)*

Others mentioned being excluded from the discussions; instead, they only received incomplete coincidental information. Even when informed of their progress or lack thereof, women could not understand all they were told, mainly as healthcare providers used medical jargon or English, a language used by the educated. Women, therefore felt excluded from the discussion of their care. Consequently, women found it difficult to ask for clarifications since they were not part of the conversation.

*“I didn’t go to school; I don’t know English, but if they (providers) speak, I notice the actions and understand…. they did not talk to me directly, and I didn’t understand their conversations except their actions, which showed me that these guys must be talking about me on something…” (Facility2_IDI_M4)*

Women did not ask questions as they felt they should listen to healthcare providers, a belief shared by many in their community. As one woman narrates:

*“Even before my arrival here (in the labour ward)… At home, my mother told me to do whatever these providers say to me because they are experienced. They will know if there is any problem. Resisting them may result in bad consequences.” (Facility 1_IDI_M7)*

### Respect and dignity during intrapartum care is not guaranteed

Women reported varied accounts of respectful and disrespectful experiences. For example, there were many “providers” who did not introduce themselves or their roles. Women suspected some of these providers were students and wished to know. Many women reported that they were treated better than their past deliveries.

#### Some women were handled with respect

Some women reported to feel respected and to receive dignified care and did not experience or witness disrespect from providers. They reported to be monitored and advised for a safe delivery at all times.

#### Experiences of mistreatment were common

Other women reported experiencing varying acts of mistreatment from healthcare providers such as non-polite language and being scolded and shouted at. As this participant details:

*“There was one [nurse] who was shouting…. when I called her, she asked me, “Don’t you understand? Take your basin, make the bed, then go to sleep!”. She undermines you and mistreats you. I felt poorly treated, with so many orders! I felt bad, was scared and frustrated; I had no option but to follow her orders.” (Facility1_IDI_M10)*

In addition, women gave accounts of being intimidated into following instructions or suffering consequences regardless of their level of pain. Non-compliant women were often threatened that their relatives would be summoned inside to witness their misbehaviours, which may lead to adverse birth outcomes. One woman narrates:

*“They tell you: ‘Don’t do like this [ early pushing]! Do you want to have a child with no brain?”.”(Facility1_IDI_M5)*

Another woman described her experience of being ignored when she had prolonged labour. As she had two prior deliveries, she could tell this was not normal labour. She narrates below:

*“Sometimes they ignore you when you call. I have given birth two times before, but this time it was different. I knew something was wrong this time as it was awful. You know your own body…thank God, after several hours, the doctor came to assess me and decided to do a caesarean section…” (Facility2_IDI_M9)*

#### Women justified and normalized acts of mistreatment

Unfortunately, women regarded mistreatment and abusive behaviours from healthcare providers as normal and even required. Women perceived that healthcare providers harshness aim to get women to comply with instructions.

*“Sometimes we mothers get confused with too much pain and behave badly. During such periods I have seen providers are doing well (being harsh) because they are saving the baby that is in the womb, now if they leave us, we may kill our babies. And there were others (providers) who pretend to be harsh just to make you do what they tell you, because other times when someone says softly do this and that some people take it easy”. (Facility2_IDI_M3)*

### Women had varying experience of support during labour

Some women mentioned getting help in different ways from the health providers or their companions (escorts). This included physical, emotional and logistical support from providers and women’s companions.

#### Women appreciated being supported during labour

Some women reported being supported, especially during the time of childbirth itself or when they were having a difficult delivery. Providers encouraged women to persevere during the second stage or held them in specific postures, and gave fluids to facilitate delivery. Several women reported that this recent delivery was a better experience compared to prior deliveries. One woman narrates her experience where she felt well supported when providers encouraged her:

*“I must say they truly helped me; when the midwife and the doctor came and helped me as I pushed. They kept encouraging me, when I had given up, they told me to have faith and said God is there; he will help you. Indeed, I delivered, God has helped me” (Facility 2 IDI_M6)*

Interestingly one woman reported that a healthcare provider applied pressure on her abdomen to support delivery and that this helped her as she narrates:

*“They were very supportive… and encouraged me to push hard. Later on, when I was getting tired one of them stepped on the bed and helped me to push her on my belly (gesturing) and the baby came out” (Facility1_IDI_M9)*

Several women preferred to have male providers as they were perceived to be more empathetic, treated women with care, and were more helpful and responsive than their female counterparts. One woman went further to say she wished for and prayed to have a male nurse during childbirth.

#### Providers’ responsiveness and empathy differed form one woman to another

Some women felt to be abandoned when they cried for help. They felt healthcare providers ignored them as troublemakers by making unnecessary cries and noise. These perceptions of abandonment were described by one first-time mother, who was less aware of the process and not knowledgeable about what to do:

*“I was calling for them to come and help me; I did not know what to do…. Had it been my second pregnancy, I would have known what to do at least… but for me, I did not even know what to do; I was just worried and frustrated…” (Facility2_IDI-M1)*

Women reported feeling blamed whenever they experienced adverse birth outcomes. One woman, who unfortunately had a stillbirth, perceived that providers implied she could have somehow saved the situation had she acted differently.

*“They (providers) said I should have come earlier, as I had a previous scar. I went to the antenatal clinic several times, and they told me to deliver in a bigger hospital, but I did not understand properly. I started bleeding at home and came here directly… I do not understand what went wrong…” (Facility2_IDI_M9)*

#### Women’s perceptions and experiences with birth companionship varied

Most women did not expect to have their relatives in the labour ward with them. They were satisfied and even justified this practice and they cited some unwritten laws about not allowing relatives and partners in the delivery ward. A few women, however, expressed their wishes to have had a familiar person during the labour process. These were young, first-time mothers, women who had operative delivery or experienced an adverse event.

Other women did not think that having a birth companion would have improved their experiences as this was an individual work.

## Discussion

We explored childbirth experiences of women as end-users of facility services, to inform an intervention to improve intrapartum care. Our main finding is that women’s experiences during childbirth vary. Sub-optimal and inefficient provider-woman communication resulted into negative experience and vice versa. Mistreatment and undue pressure on clients were commonly reported and support or the lack thereof was standard for all clients regardless their needs. Many women seemed to have low expectations and thus low motivation to complain. We could not find a pattern of who and why some reported negative experiences while others did not.

Several factors are known to affect women’s childbirth experiences within the facility setting. Sub-optimal communication during admission result in a strained provider-woman relationship, which affect collaborative interactions during labour and childbirth [6, 10, 20, 33, 34]. In addition, women in the study had low education, were of low socio-economic status and had low health literacy, and were not able to demand quality care and received the offered care as it is [6, 12, 20, 33, 35]. Furthermore, women come to the labour ward uncertain, and apprehensive; often, they meet the providers for the first time and not able to start a conversation [3, 33]. The society has already prepared women to be obedient to providers, as they have superiority in their knowledge of the labour process [7, 10, 12, 33, 34, 36]. Often women relinquish decision-making role to providers due to their low perceived self-efficacy [8, 20, 37, 38].

Similar to other studies, we found mistreatment was common and providers use this as means to ensure compliance from women [1, 3, 7, 8, 39]. Furthermore, mistreatment acts were accepted by women as health providers’ reaction towards a constrictive working environment [2, 3, 7, 10, 12, 34, 40].

There was low awareness and expectation on birth companionship in both hospitals, despite the documented good outcomes of this practice [14, 15, 17, 41]. A pilot intervention in the country urged the Tanzanian Ministry of Health to develop a guideline on *Gender and Respectful Care Mainstreaming and Integration Across Reproductive Maternal Newborn Child and Adolescent Healthcare services, that urged facilities to incorporate respectful maternity care and birth companionship* [42]. There are several barriers that hinder effective uptake of birth companionship, including non-permissive infrastructures, low provider acceptance, fear of stigma, social and gender norms mystifies childbirth as a women’s matter, and misconceptions of regulations that bar relatives especially husbands to be in the delivery ward which is conceived as a woman’s space [15, 17].

Women who reported good experiences praised providers who were compassionate and upheld their professional value. It is possible that some of these “good” experiences may be due to low expectations, in such a way that any care is perceived as good. As for the woman who received fundal pressure, she appreciated the act, not understanding that it was potentially harmful. Fortunately, women did not report to observe or encounter physical abuse.

We report on several interesting findings, that did not confer to our expectations. For example, there was a preference to male providers contrary to traditional beliefs that the delivery ward is mostly a women’s place [17, 20, 35], perhaps indicating that women’s perceptions and beliefs are changing to become permissive on good versus the normal. This is an area that need to be explored further.

### Implication for practice

The study findings highlight the importance to listen to women’s experiences of childbirth care, hear what is important, useful or painful to them and what is not. Such voices can guide interventions for quality improvement within the healthcare setting, a practice that is not common.

Empowering women with health literacy and confidence, demystifying social norms and having a dialogue on gender and social norms can empower women to understand and demand quality care, including being important players in the discussion around their childbirth care. Several strategies can be used to achieve this; utilize the antenatal care visits to introduce dialogues that tackle myth and misconceptions on childbirth process, as well as prepare women to have better relationship with labour ward staff, before being in labour. In addition, strategies to initiate meaningful discourse at the community level; address the societal norms and myths on provider’s superiority and increase awareness of beneficial practices like birth companionship are important. Such practices will empower pregnant women, reduce their vulnerability to mistreatments and their fear and uncertainties of the childbirth process.

Capacity building of health providers, both in-service and pre-service training should be strengthened to have competent and confident providers who will not shy away from communicating and supporting women in the best way possible.

### Implication for research

Understanding research gaps from women’s perspective and childbirth experiences will improve the research relevance and its impact. For example, findings from this study were used to inform the co-design of the ALERT intervention package, which considered women’s experiences. The ALERT intervention package includes providers competency-based training, mentoring, and quality improvement in areas that were sources of negative experiences such as inefficient communication and non-respective care.

Future research should also look into what causes women to report different experiences and how to make the health system deliver to all women equally. Outside the health facility, we need research to explore knowledge, norms and beliefs of childbirth and the support around. Co-design process with women during antenatal care can also help to have better linkage from the antenatal clinic to the labour ward and harness this window of opportunity for better pregnancy and childbirth experiences.

Good experiences from the study area can be built on and used as learning points to increase the quality of care offered within the facilities.

### Implication for planning and policy making

Tanzania has several policies and guidelines that aim to improve women’s status and ensure quality healthcare services. Dissemination and implementation of these policies need to be strengthened.

### Strength and limitations

To our knowledge, this is the first study that used the women’s experiences to inform an intervention co-design in Tanzania. Findings were validated and potential interventions co-designed with women.

Our limitations were that we only reported on women’s experiences. Providers’ and community member’s narrations which will be presented in different publications. We interviewed postpartum women while they were in the health facility, which may have reduced their freedom to respond [9]. However, women shared their negative and positive experiences, so we believe the location or timing of interviews did not infringe the findings.

## Conclusion

We reported that women’s’ experiences of childbirth varied: the experiences of communication, respectful care and support varied from one woman to the next. Indicating the care provided to be unequitable and not always cantered on the women’s needs. The planned co-designed quality improvement intervention should, therefore, include i) empowering women to voice their individual needs for emphatic interaction with healthcare providers, ii) having continuous dialogue with women to de-normalize disrespectful care and broken communication, and iii) supporting healthcare care workers to support women on communication and interaction that is meaningful.

### Supplementary Information

**Supplementary Material 1.**

**Supplementary Material 2.**

## Data Availability

No datasets were generated or analysed during the current study.

## Abbreviation

WHO: World Health Organization
