# Supplementary material for: Exploring women’s experiences of care during hospital childbirth in rural Tanzania: a qualitative study
Source: BMC Pregnancy Childbirth. 2024 Apr 19;24:290. doi: 10.1186/s12884-024-06396-0 (PMC11027221; doi:10.1186/s12884-024-06396-0)
Supplement: Supplementary file 1 — Supplementary Material 1. [file 12884_2024_6396_MOESM1_ESM.pdf]

TOOL 1 INTERVIEW WITH MOTHERS:

Tool 1: Overview of topics and questions to women

1. Introduction to interview focus and process: Place of childbirth, date, status of child

Background information on mother and child

2. Childbirth experience of mother: Please tell me about your childbirth from the beginning to the end? Tell me about To your first contact with a health staff upon arriving at the facility.
3. Communication with staff - responsiveness *NOTE: Try to reconstruct how many health staff the woman has been in contact with:*

- Who was the first contact at the maternity ward?
- What questions were you asked, and how did you respond?
- What was difficult for you to talk about?
- Did they call you by name? If yes, what name?
- Do you recall if they were nurses/midwives/doctors?
- Did staff come when you called them? If yes, how did they react to your calls?
- How did they talk to you about any procedures done on you or your baby?
- How did they describe possible complications to you?
- How were you encouraged during labour? During pushing?
- Did providers talk about you in your presence? If yes, how did they talk about you? How did that make you feel?
- Were staff disrespectful to you? In what ways? Tell me what happened.
- Were staff complaining about something you did/did not?
- Were you slapped? Yelled at?

4. Support from a companionon . Who chose to accompany you to the facility? Who else was considered? Why?

- What kind of support did the companion/s offer to you? *Probe: Emotional support, physical support; logistical support, communication and interaction*

- What would you have preferred to be different? *Probe on: The companion; support/ Lack of support; skill of companion*
- Were there any barriers that you experienced with a companion? Willingness to change from companion, health staff?
- Can you describe whether the environment was supportive/allowing companionship?
- How would the physical setting have needed to be improved to allow for that?

5. Compare expectations to experience. How would you compare your expectations of childbirth at this facility to your experience? What did you hope for? What did you expect to happen, although you hoped it wouldn't?

6. **Final question.** What have we not yet talked about? Please add.
