# Supplementary material for: Exploring women’s experiences of care during hospital childbirth in rural Tanzania: a qualitative study
Source: BMC Pregnancy Childbirth. 2024 Apr 19;24:290. doi: 10.1186/s12884-024-06396-0 (PMC11027221; doi:10.1186/s12884-024-06396-0)
Supplement: Supplementary file 2 — Supplementary Material 2. [file 12884_2024_6396_MOESM2_ESM.docx]

Supplementary files


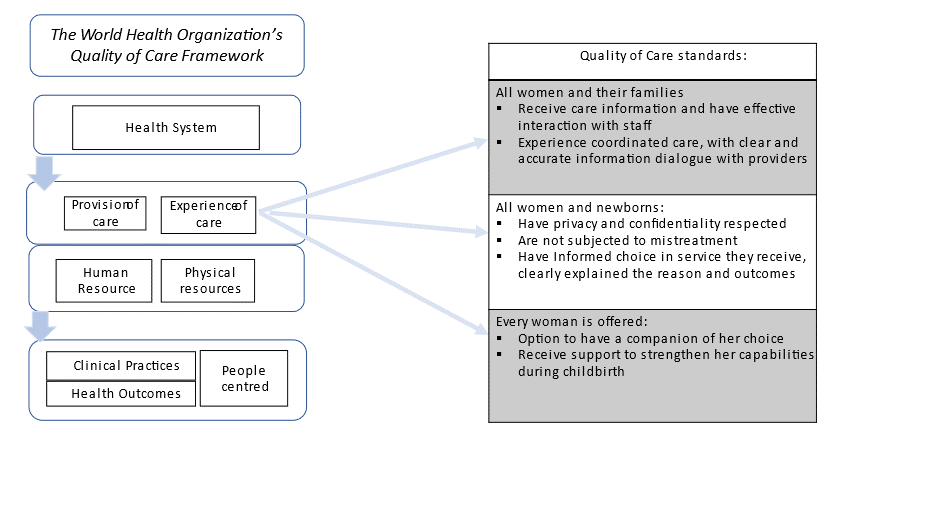


Supplementary figure. The World Health Organization framework for quality of care for women and newborns with quality standards [4, 5, 37]
